# Association between anemia and hyperuricemia: results from the Korean National Health and Nutrition Examination Survey

**DOI:** 10.1038/s41598-019-55514-y

**Published:** 2019-12-13

**Authors:** Yeonghee Eun, Kyung-Do Han, Da Hye Kim, In Young Kim, Eun-Jung Park, Seulkee Lee, Hoon-Suk Cha, Eun-Mi Koh, Jaejoon Lee, Hyungjin Kim

**Affiliations:** 1 Department of Medicine, Samsung Medical Center, Sungkyunkwan University School of Medicine, Seoul, Korea; 2 Department of Biostatistics, College of Medicine, The Catholic University of Korea, Seoul, Korea; 3 Department of Medicine, National Police Hospital, Seoul, Korea; 4 Department of Medicine, National Medical Center, Seoul, Korea

**Keywords:** Risk factors, Comorbidities

## Abstract

Hyperuricemia and anemia share several comorbidities, but the association between the two conditions remains unclear. The purpose of this study was to investigate the association between hyperuricemia and anemia. Data of 10794 subjects from the Korean National Health and Nutrition Examination Survey conducted in 2016–2017 were analyzed using multivariate logistic regression analyses. An association between anemia and hyperuricemia was not evident in subjects without chronic kidney disease (CKD). In patients with CKD, anemia increased the risk of hyperuricemia by 2-fold. This association remained significant when adjusting for the glomerular filtration rate. In subgroup analyses, the association of anemia with hyperuricemia was significant in subjects aged ≥65 years, and in those with diabetes or hypertension. Subgroup analyses of CKD patients showed similar results. In the current study using data from Korean representative samples, anemia in subjects with CKD was associated with a 2-fold increase in the risk of hyperuricemia, which remained significant even after adjustment for renal function.

## Introduction

Hyperuricemia is characterized by abnormally elevated levels of serum uric acid. Hyperuricemia is the best-known risk factor for gout, but it is also a risk factor for hypertension, diabetes, and chronic kidney disease (CKD)^1–3^. In addition, it increases the risk of coronary heart disease independent of traditional cardiovascular risk factors^4–6^. According to the US National Health and Nutrition Examination Survey (NHANES) data, the prevalence of hyperuricemia in American adults was 21% in 2007–2008, when was 3.2% more than that in 1988–1994^7^. An analysis of the 2016 Korean National Health and Nutrition Examination Survey (KNHANES) data showed that the age-standardized prevalence of hyperuricemia in the general Korean population was 11.4%^8^. Considering the high prevalence of hyperuricemia, it is important to consider its effects on various diseases. A number of studies have been conducted on the association between hyperuricemia and its comorbidities^9–11^.

Anemia is associated with poorer prognosis in patients with cardiovascular diseases^12,13^. The global anemia prevalence in 2010 was 32.9%^14^. Hyperuricemia and anemia may be related in terms of sharing comorbidities such as CKD and cardiovascular disease. However, to the best of our knowledge, no studies have been conducted on the relationship between the two conditions.

The literature on the relationship between gout and anemia is sparse. One study investigated the association between gout and anemia. In a 9-year follow-up cohort study of about 10000 patients, however, the risk of gout was doubled in patients with anemia compared to that in those without anemia^15^. Interestingly, anemia increased the risk of gout even after adjusting for serum urate levels and kidney function.

This study aimed to investigate the association between hyperuricemia and anemia using data from KNHANES VII, which is a representative sample of the Korean population. Since CKD has a significant effect on both hyperuricemia and anemia, we performed this analysis by dividing groups according to the presence of CKD.

## Results

### Baseline characteristics of the study population

Of the subjects who participated in the study, 1302 (12.1%) had hyperuricemia. The mean uric acid levels were 7.5 and 4.8 mg/dL in the groups with and without hyperuricemia, respectively. Table 1 shows the general characteristics of the study population with and without hyperuricemia. Subjects with hyperuricemia had a lower glomerular filtration rate and a higher proportion of CKD than those without it. Conversely, the hemoglobin levels were higher in participants with hyperuricemia, and the proportion of anemia was lower. The overall characteristics of the non-CKD subjects, which accounted for most of the participants, were similar to those of the entire population. Unlike subjects without CKD, those with CKD and hyperuricemia were older, less likely to be male, and less educated, although these differences were not statistically significant. There was no statistically significant difference in eGFR according to the presence of hyperuricemia among CKD subjects. In the CKD group, the prevalence of anemia was higher in hyperuricemic subjects (43.2%) than in non-hyperuricemic subjects (28.6%).

**Table 1:** Characteristics of study population according to hyperuricemia.

	Total (n = 10794)			Non-CKD (n = 10455)			CKD (n = 341)		
	Non-hyperuricemic	Hyperuricemic	*p*	Non-hypeuricemic	Hyperuricemic	*p*	Non-hyperuricemic	Hyperuricemic	*p*
	(n = 9494)	(n = 1302)		(n = 9312)	(n = 1143)		(n = 182)	(n = 159)	
Age, years	47.0 ± 0.3	44.4 ± 0.6	<0.0001	46.7 ± 0.3	42.2 ± 0.5	<0.0001	68.7 ± 0.9	69.5 ± 1.2	0.5632
Sex, male	46.0 (0.5)	76.7 (1.3)	<0.0001	46.0 (0.5)	79.6 (1.3)	<0.0001	49.6 (4.3)	42.4 (4.4)	0.2407
Urban residence	85.0 (1.7)	86.0 (1.9)	0.5200	85.1 (1.7)	86.6 (1.9)	0.3123	78.6 (4.2)	78.4 (4.0)	0.9723
Occupation	64.4 (0.7)	67.5 (1.7)	0.0759	64.8 (0.7)	71.0 (1.8)	0.0008	33.1 (4.3)	26.4 (3.7)	0.2197
Income (lowest quartile)	15.2 (0.7)	15.1 (1.3)	0.9953	14.8 (0.7)	11.9 (1.3)	0.0298	40.4 (4.1)	52.8 (4.6)	0.0445
Low education level^a^	23.5 (0.8)	19.5 (1.3)	0.0007	23.1 (0.8)	15.8 (1.2)	<0.0001	56.3 (4.1)	62.0 (4.4)	0.3324
Energy intake, kcal/day	1963 ± 14	2138 ± 43	<0.0001	1968 ± 14	2194 ± 46	<0.0001	1611 ± 60	1531 ± 66	0.3431
Alcohol consumption			<0.0001			<0.0001			0.6519
None	23.1 (0.6)	15.8 (1.1)		22.6 (0.6)	12.5 (1.1)		59.4 (4.4)	55.2 (4.5)	
<30 g/day	68.8 (0.6)	67.8 (1.5)		69.2 (0.6)	70.0 (1.6)		36.4 (4.3)	41.8 (4.5)	
≥30 g/day	8.2 (0.3)	16.4 (1.3)		8.2 (0.3)	17.5 (1.4)		4.2 (2.0)	3.0 (1.5)	
Smoking			<0.0001			<0.0001			0.0609
Never	61.8 (0.6)	43.6 (1.6)		61.9 (0.6)	42.2 (1.6)		56.7 (4.1)	61.2 (4.7)	
Former	18.3 (0.4)	24.4 (1.4)		18.1 (0.5)	24.6 (1.5)		32.7 (4.0)	21.5 (3.5)	
Current	20.0 (0.6)	32.0 (1.5)		20.1 (0.6)	33.3 (1.6)		10.6 (2.4)	17.3 (3.5)	
Physical activity^b^	47.0 (0.7)	51.8 (1.7)	0.0056	47.2 (0.8)	53.6 (1.8)	0.0005	35.4 (4.2)	31.0 (4.5)	0.4795
BMI, kg/m^2^	23.7 ± 0.1	25.9 ± 0.1	<0.0001	23.6 ± 0.1	26.0 ± 0.1	<0.0001	24.4 ± 0.3	24.8 ± 0.4	0.3550
WC, cm	81.3 ± 0.2	88.3 ± 0.3	<0.0001	81.2 ± 0.2	88.4 ± 0.4	<0.0001	86.4 ± 0.9	87.4 ± 1.0	0.4672
Systolic BP, mmHg	116.9 ± 0.2	121.8 ± 0.5	<0.0001	116.7 ± 0.2	121.2 ± 0.5	<0.0001	128.6 ± 1.5	128.3 ± 1.5	0.8769
Diastolic BP, mmHg	75.4 ± 0.2	79.4 ± 0.4	<0.0001	75.5 ± 0.2	80.1 ± 0.4	<0.0001	73.4 ± 0.9	71.7 ± 1.1	0.2202
Fasting glucose, mg/dL	99.4 ± 0.3	100.5 ± 0.6	0.0784	99.2 ± 0.3	99.4 ± 0.6	0.6738	113.6 ± 3.2	112.9 ± 3.0	0.8671
Total C, mg/dL	192.3 ± 0.5	201.3 ± 1.4	<0.0001	192.5 ± 0.5	203.1 ± 1.4	<0.0001	177.7 ± 3.2	180.7 ± 4.0	0.5368
TG^c^, mg/dL	106.9 (105.3–108.5)	155.7 (148.5–163.2)	<0.0001	106.7 (105.0–108.3)	157.6 (149.8–165.8)	<0.0001	128.9 (120.1–138.2)	135.3 (125.1–146.4)	0.3596
eGFR^d^, mL/min/1.73 m^2^	97.7 ± 0.3	88.4 ± 0.6	<0.0001	98.4 ± 0.3	91.9 ± 0.5	<0.0001	48.8 ± 1.3	47.5 ± 1.0	0.4393
Hemoglobin, g/dL	14.11 ± 0.02	14.94 ± 0.05	<0.0001	14.13 ± 0.02	15.12 ± 0.05	<0.0001	13.12 ± 0.14	12.80 ± 0.16	0.1342
Uric acid, mg/dL	4.77 ± 0.01	7.54 ± 0.03	<0.0001	4.77 ± 0.01	7.52 ± 0.03	<0.0001	5.11 ± 0.10	7.66 ± 0.10	<0.0001
hs-CRP^c^, mg/L	0.67 (0.66–0.69)	0.95 (0.90–1.01)	<0.0001	0.67 (0.65–0.69)	0.94 (0.88–1.00)	<0.0001	0.93 (0.80–1.09)	1.06 (0.89–1.27)	0.2774
Obesity^e^	31.6 (0.7)	56.3 (1.6)	<0.0001	31.5 (0.7)	57.1 (1.7)	<0.0001	40.6 (3.9)	47.3 (4.3)	0.2457
Abdominal obesity^f^	25.5 (0.7)	46.9 (1.6)	<0.0001	25.2 (0.7)	46.4 (1.7)	<0.0001	48.8 (4.3)	52.7 (4.3)	0.4964
Hypertension^g^	25.2 (0.6)	38.3 (1.7)	<0.0001	24.6 (0.6)	34.7 (1.7)	<0.0001	69.6 (4.0)	80.2 (3.8)	0.0708
Diabetes melitus^h^	10.3 (0.4)	10.6 (1.0)	0.8186	9.9 (0.4)	7.8 (0.9)	0.0371	44.8 (4.1)	43.6 (4.5)	0.8458
Hyperlipidemia^i^	19.5 (0.5)	23.5 (1.4)	0.0034	19.3 (0.5)	22.7 (1.5)	0.0162	37.8 (4.0)	33.7 (4.8)	0.5263
Chronic kidney disease^j^	1.3 (0.1)	7.9 (0.7)	<0.0001						
Anemia^k^	8.0 (0.3)	5.6 (0.6)	0.0014	7.7 (0.3)	2.4 (0.4)	<0.0001	28.6 (3.9)	43.2 (4.3)	0.0164

Data are presented as weighted mean ± standard error (SE) or weighted percentage (SE). ^a^Low education level means middle school graduate or less. ^b^Physical activity is defined in accordance with the WHO recommendations, which refers to more than 150 minutes of moderate-intensity aerobic physical activity throughout the week, or more than 75 minutes of vigorous-intensity aerobic physical activity throughout the week, or an equivalent combination of moderate- and vigorous-intensity activity. ^c^TG and hs-CRP values are presented as geometric mean (95% confidence interval). ^d^An eGFR calculated from serum creatinine using an isotope dilution mass spectrometry (IDMS) traceable Modification of Diet in Renal Disease (MDRD) Study equation. ^e^Obesity is defined as BMI 25 kg/m^2^ or higher according to the proposed classification of weight by BMI in adult Asians. ^f^Abdominal obesity is defined as a WC in men of 90 cm or more and in women of 85 cm or more in Korea. ^g^Hypertension is defined as a systolic BP > 140 mmHg, or a diastolic BP > 90 mmHg, or taking antihypertensive medication. ^h^Diabetes mellitus is defined as a fasting blood glucose level > 126 mg/dL, or previous history of diagnosis by doctor, or taking hypoglycemic agent or insulin. ^I^Hyperlipidemia is defined as a fasting total C ≥ 240 mg/dL or taking lipid lowering agent. ^j^Chronic kidney disease is defined as an eGFR <60 mL/min/1.73 m^2^. ^k^Anemia is defined as hemoglobin <12 g/dL for women and <13 g/dL for men. BMI, body mass index; WC, waist circumference; BP, blood pressure; C, cholesterol; TG, triglyceride; eGFR, estimated glomerular filtration rate; hs-CRP, high-sensitivity C-reactive protein.

### Association between anemia and hyperuricemia

**Figure 1:**
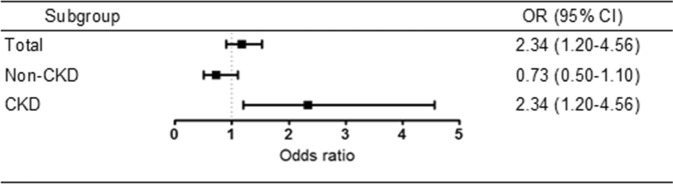
Forest plots of the odds ratio of hyperuricemia according to anemia. CKD, chronic kidney disease.

We performed a multivariate logistic regression analysis to determine the association between anemia and hyperuricemia (Table 2). In non-CKD subjects, the risk of hyperuricemia was significantly lower when anemia was present (OR 0.68 in models adjusted for age, sex, BMI, smoking, alcohol drinking, physical activity, income, and education levels; 95% confidence interval 0.47–0.99), but the difference was not statistically significant when hypertension and diabetes were further adjusted. In CKD subjects, the risk of hyperuricemia was 2-fold higher when anemia was present (Fig. 1). This association persisted, independent of adjustment, and remained significant even after adjustment of eGFR, a value representing the degree of renal function. When CKD was analyzed in two groups based on an eGFR of 30 mL/min/1.73 m^2^, the statistical significance of the association between anemia and hyperuricemia was maintained in CKD stage 3 group (eGFR 30–60 mL/min/1.73 m^2^). In the group with CKD less than stage 2 (eGFR < 30 mL/min/1.73 m^2^), anemia and hyperuricemia tended to correlate, although the small number of patients did not allow us to obtain statistical significance (Supplementary Table S1).

**Table 2:** Multivariate logistic regression analysis of the association between anemia and hyperuricemia.

Subgroup	OR (95% CI)				
	Model 1	Model 2	Model 3	Model 4	Model 5
Total	0.68 (0.54–0.87)	1.18 (0.93–1.51)	1.42 (1.11–1.82)	1.44 (1.12–1.84)	1.18 (0.90–1.53)
Non-CKD	0.29 (0.21–0.42)	0.57 (0.39–0.83)	0.68 (0.47–0.99)	0.69 (0.48–1.01)	0.73 (0.50–1.10)
CKD	1.90 (1.12–3.23)	1.91 (1.12–3.24)	2.13 (1.20–3.78)	2.24 (1.23–4.07)	2.34 (1.20–4.56)

ORs and 95% CIs for the presence of hyperuricemia. Model 1 is non-adjusted model. Model 2 is adjusted for age and sex. Model 3 is adjusted for all variables in model 2 plus BMI, smoking, alcohol drinking, physical activity, income, education level. Model 4 is adjusted for all variables in model 3 plus hypertension and diabetes. Model 5 is adjusted for all variables in model 4 plus glomerular filtration rate. OR, odds ratio; 95% CI, 95% confidence interval; CKD, chronic kidney disease.

**Figure 2:**
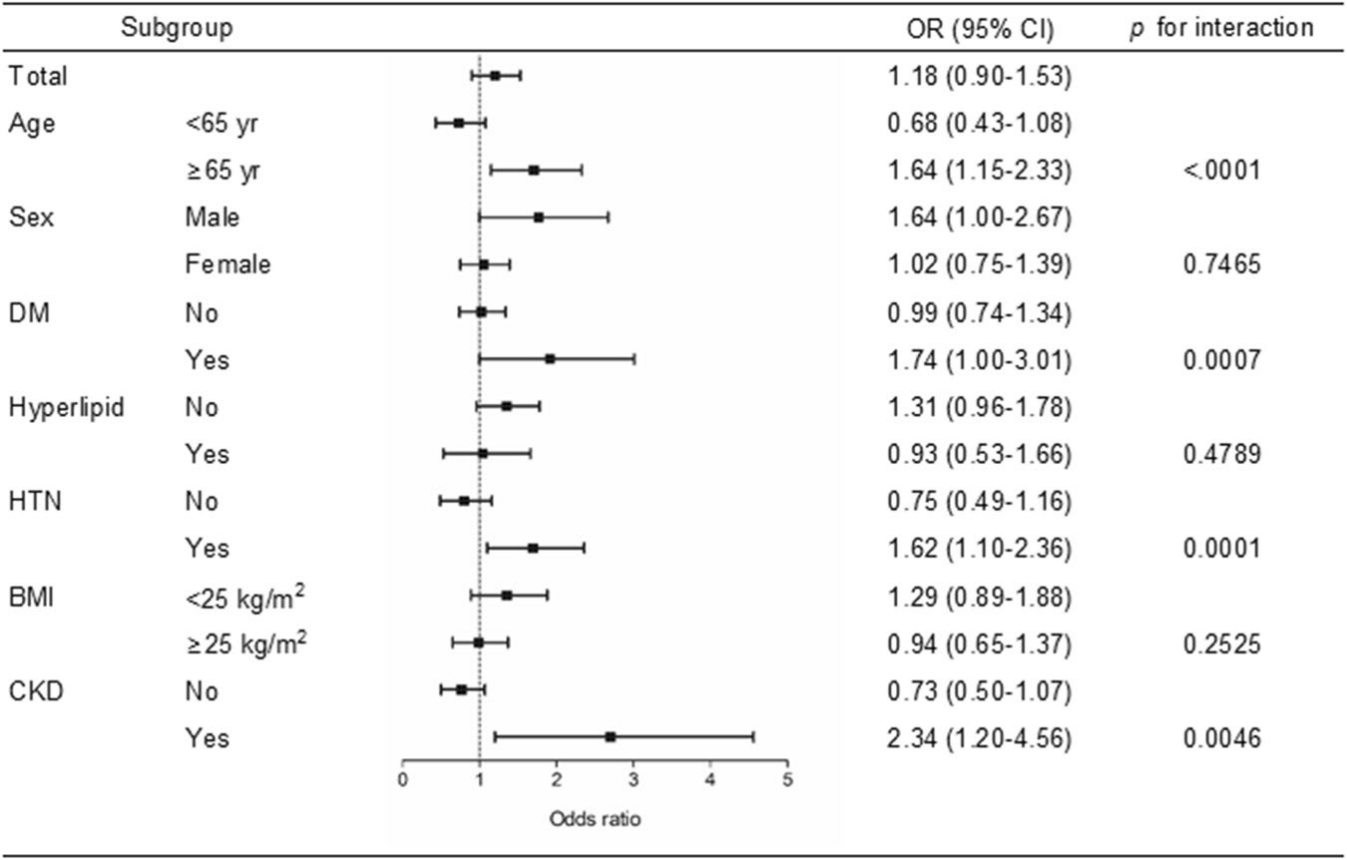
Forest plots including subgroup analyses of the odds ratio of hyperuricemia in relation to anemia. DM, diabetes mellitus; Hyperlipid, hyperlipidemia; HTN, hypertension; BMI, body mass index; CKD, chronic kidney disease.

Subgroup analyses were performed to determine whether the association between anemia and hyperuricemia varies according to participant characteristics (Fig. 2). The association between anemia and hyperuricemia was evident in subjects aged ≥65 years, in those with diabetes, and in those with hypertension.

**Figure 3:**
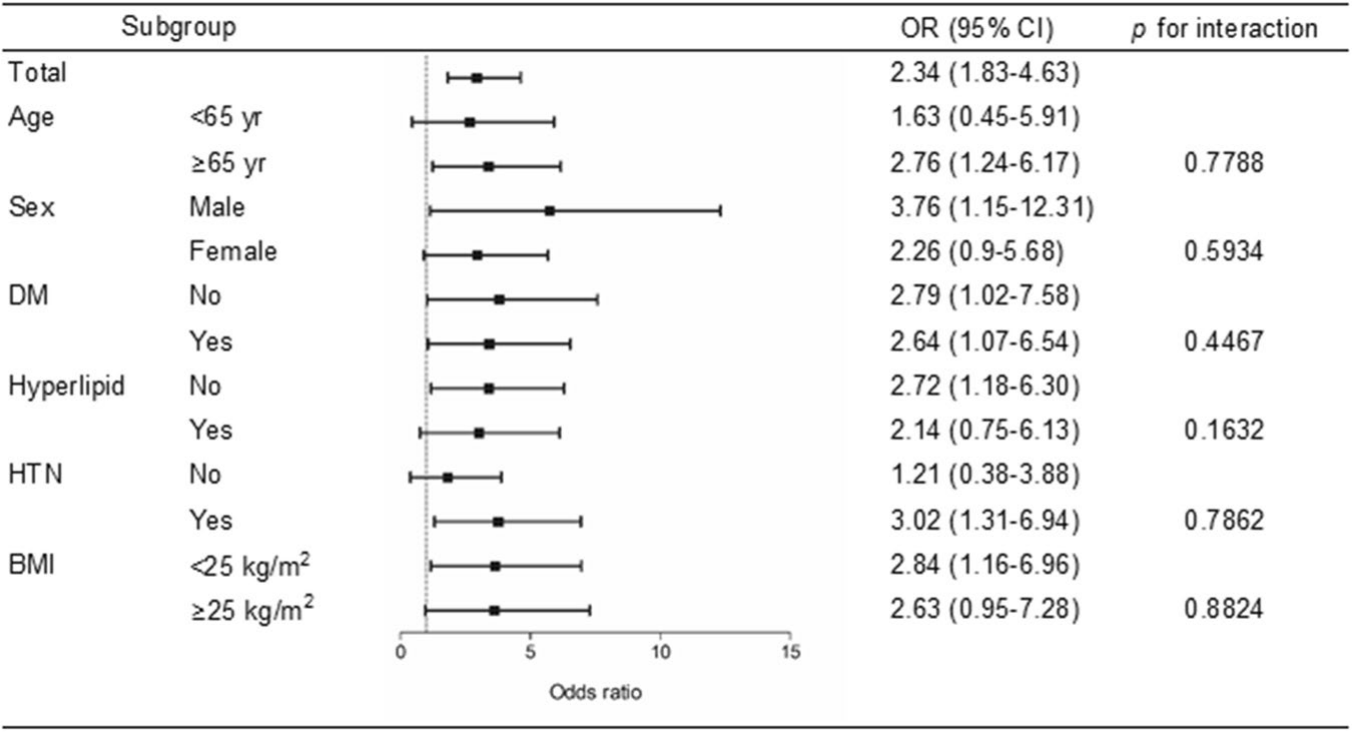
Forest plots including subgroup analyses in subjects with chronic kidney disease of the odds ratio of hyperuricemia in relation to anemia. DM, diabetes mellitus; Hyperlipid, hyperlipidemia; HTN, hypertension; BMI, body mass index.

In the subgroup analyses of CKD patients, the association between anemia and hyperuricemia remained significant in most subgroups, although this association was more pronounced in older and in hypertensive patients (Fig. 3).

## Discussion

In this study, we analyzed the association between anemia and hyperuricemia using representative sample data from the noninstitutionalized civilian Korean population. The association between anemia and hyperuricemia was distinct depending on the presence of CKD. In subjects with CKD, the risk of hyperuricemia was doubled in the presence of anemia, and this association remained significant after adjustment for renal function. In those without CKD, anemia and hyperuricemia were inversely correlated. The association between anemia and hyperuricemia varied depending upon the characteristics of the subjects. Anemia increased the risk of hyperuricemia in people aged ≥65 years, and in those with diabetes or hypertension. In CKD subjects, anemia generally increased the risk of hyperuricemia, but the association was more pronounced in older and in hypertensive participants.

The baseline characteristics of the study population according to the presence of hyperuricemia were significantly different between the CKD and non-CKD groups. Sex, alcohol consumption, physical activity, hypertension, hyperlipidemia and obesity, which are well known risk factors of hyperuricemia, differed in the presence of hyperuricemia in the non-CKD group but not in the CKD group^8,16–18^. These results indicated that CKD itself is the most important factor in the development of hyperuricemia in CKD patients.

Unlike other variables, which showed no significant differences in the CKD group, anemia had significantly higher prevalence in hyperuricemic subjects than in non-hyperuricemic subjects. In a multivariate logistic regression analysis that examined the association between anemia and hyperuricemia, anemia was associated with a 2-fold increase in the risk of hyperuricemia in CKD subjects. We propose two possible explanations for these things. First, anemia is more common in patients with advanced stages of CKD, and hyperuricemia is more common as CKD progresses, which may explain the coexistence of hyperuricemia and anemia as a result of poor renal function^19–21^. However, a significant association between anemia and hyperuricemia even after adjustment for eGFR, representing renal function, suggests that there is a direct relationship between the two conditions, and not that they are merely coexisting conditions due to deterioration of renal function. Second, anemia leads to an increase in oxidative stress, which may result in hyperuricemia. Several studies have reported high oxidative stress levels in patients with renal disease^22–24^. Oxidative stress affects the cellular structure and function of erythrocytes and is involved in the development of anemia^24^. Conversely, anemia itself in CKD patients may increase oxidative stress. This has been indirectly demonstrated by studies in which serum levels of aldehydic lipid peroxidation products were reduced when renal anemia was corrected through erythropoietin treatment^25^. Hypoxia and impaired oxidative metabolism are associated with elevated uric acid levels^26,27^. Hypoxia induces accumulation of the uric acid precursors hypoxanthine and xanthine, activating xanthine dehydrogenase and xanthine oxidase^28,29^.

The number of CKD patients included in this study is much smaller than the number of non-CKD subjects. According to a study on CKD prevalence in Korea, the prevalence of CKD above stage 3, corresponding to the CKD definition of glomerular filtration rate <60 mL/min/1.73 m^2^, was 2.5%, which is lower than the global mean prevalence of 10.6% calculated from meta-analysis^30,31^. The prevalence of CKD in this study was about 3.2%, which reflects the prevalence of the general Korean population. Despite the small number of CKD patients, a clear association between hyperuricemia and anemia was identified.

The results of this study showed that anemia and hyperuricemia are inversely correlated in non-CKD subjects, contrary to that in CKD subjects. This may reflect nutritional status. In hyperuricemic subjects, higher energy intake, higher BMI, and higher obesity prevalence than non-hyperuricemic subjects appeared to indicate that hyperuricemic subjects had better nutrition than non-hyperuricemic subjects^32^. Since malnutrition is an important factor contributing to the development of anemia, such differences in nutrition may have affected the inverse correlation between hyperuricemia and anemia^33,34^. Alcohol consumption has opposite effect on anemia and hyperuricemia, which negatively affects anemia but increases hyperuricemia^35–37^. Considering that obesity is associated with chronic, low-grade inflammation, the prevalence of anemia of inflammation may be higher in obese persons^38,39^. A study of 14848 NHANES III participants in the US confirmed that overweight and obese persons did not have a higher prevalence of anemia than normal-weight persons^40^. The pathogenesis of anemia of inflammation contributes to dysregulation of iron homeostasis, impaired proliferation of erythroid progenitor cells, and blunted erythropoietin response^41^. Obesity can cause hypoferremia owing to changes in iron homeostasis but does not affect red-cell survival or erythropoiesis^42^. Therefore, the prevalence of anemia of inflammation may not be high in obese persons. Elevation of erythropoietin levels associated with hypoxia due to obstructive sleep apnea whose incidence increases in obesity or cellular hypoxia due to adipose tissue expansion in obese persons may also be possible explanations^43,44^.

In the non-CKD group, individuals with hyperuricemia were younger and more physically active and had higher C-reactive protein levels than those without. The link between inflammation and uric acid has been demonstrated in previous studies^45–47^. Regardless of hyperuricemia being a cause or a consequence of inflammation, this study cannot clearly identify causal relationships due to the limitations of cross-sectional studies. However, it is reasonable to understand that hyperuricemia induces inflammation based on the recent report that proinflammatory NF-KB signaling pathway was activated when human hepatoma HepG2 cells were exposed to uric acid^45^.

The difference in the association between anemia and hyperuricemia according to presence of CKD may be due to differences in the mechanism of anemia with or without CKD. Anemia observed in CKD is attributed to various factors such as decreased erythropoietin production, shortened erythrocyte life span, inflammation, and increased blood loss^48^. According to a systemic analysis of global anemia burden from 1990 to 2010, the most common type of anemia was iron deficiency anemia^14^. Subsequently, iron deficiency anemia may be the most common type in non-CKD subjects, which accounts for most of the population of this study. In previous studies, positive correlations between ferritin, iron, and serum urate levels have been demonstrated^49,50^. Therefore, hyperuricemia is less likely to occur with anemia in non-CKD subjects, in whom iron deficiency anemia is a major component. In addition, as described earlier, CKD itself plays an important role in increasing oxidative stress, so that oxidative stress induced by anemia in non-CKD patients may be insufficient to cause hyperuricemia.

In subgroup analyses, the association between anemia and hyperuricemia was more pronounced in older subjects, and in subjects with hypertension or diabetes. Old age, hypertension, and diabetes are variables associated with anemia and hyperuricemia^1,10,51–54^. Therefore, the prevalence of hyperuricemia and anemia may be higher in patients with these characteristics. In the subgroup analysis of CKD subjects, the association between anemia and hyperuricemia was clearly observed, regardless of the characteristics of the subjects. However, this association was more evident in older subjects, and in those with hypertension.

There are several limitations to our study. First, this was a cross-sectional study. Hence although the association between anemia and hyperuricemia could be confirmed, a causal relationship cannot be verified. Second, since KNHANES did not contain detailed information about underlying diseases that might affect anemia or medication taken by subjects, we could not adjust for treatment of anemia or hyperuricemia in analyses. Third, the causes of anemia vary, but KNHANES does not include information such as erythropoietin level, iron state, or vitamin B12 level, which can be used to identify the cause of anemia. Therefore, no analysis of the association of hyperuricemia with the cause of anemia was performed in this study. Fourth, the small number of CKD patients did not allow discrimination between CKD stages. Although eGFR was adjusted to reflect the renal function, further studies including more CKD patients are needed to determine whether the association between anemia and hyperuricemia will increase as the CKD stage progresses. Finally, the prevalence of anemia and hyperuricemia may vary according to race or characteristics of the subjects, thus generalizability may be limited in applying the results of this study. Further studies are needed to confirm whether the relationship between anemia and hyperuricemia is maintained in a population of different races or characteristics and to identify the mechanism by which anemia contributes to hyperuricemia. Despite these limitations, this study is valuable because it suggests a direct association between hyperuricemia and anemia.

Using data from Korean representative samples, anemia in CKD subjects is associated with a 2-fold increase in the risk of hyperuricemia compared to that in non-CKD subjects. And this association remains significant even after adjustment for renal function. These results demonstrate that the two conditions may have a direct relationship, and are not merely coexisting conditions.

## Materials and Methods

### Study population

In this study, data from the seventh KNHANES, conducted by the Korean Centers for Disease Control and Prevention (KCDC) in 2016–2017 were analyzed. KNHANES is designed to assess the health and nutritional status of a representative sample of the noninstitutionalized civilian Korean population. The survey consisted of three parts: a health interview, a health examination, and a nutrition survey. Trained interviewers and laboratory technicians performed the survey. Serum uric acid concentrations were included in the survey for the first time in 2016.

Of the 16277 participants, 3377 were excluded based on age (<19 years). We also excluded 2106 participants who had any missing data for variables of interest. Overall, this study included 10794 subjects.

### Collection of data

Demographic and socioeconomic data were collected, including age, sex, residential area, household income, and educational levels. Health-related habits such as alcohol consumption, smoking, and physical activity and the prevalence of comorbidities such as hypertension, diabetes, and hyperlipidemia were also investigated. Height, body weight, waist circumference, and blood pressure were measured and laboratory tests such as blood cell counts, fasting glucose, lipid profile, creatinine, uric acid and high sensitivity C-reactive protein (hsCRP) were performed in the survey. Body mass index (BMI) was calculated as weight in kilograms divided by height in meters squared.

Anemia was defined in accordance with the World Health Organization (WHO) criteria^55^, as hemoglobin of <13 g/dL in men and <12 g/dL in women. Hyperuricemia was defined as serum uric acid level ≥7.0 mg/dL in men and ≥6.0 mg/dL in women. CKD was defined as an estimated glomerular filtration rate (eGFR) <60 mL/min/1.73 m^2^.

### Statistical analysis

Student’s *t*-test, chi-square test, and Cochran–Armitage trend test were used to compare baseline characteristics, such as demographic, socioeconomic, anthropometric, and laboratory variables, according to the presence or absence of hyperuricemia. Since CKD has significant effects on both hyperuricemia and anemia, baseline characteristics were further analyzed according to the presence of hyperuricemia in CKD and non-CKD subjects. We performed a weighted multivariate logistic regression analysis to assess the association between anemia and hyperuricemia. All statistical analyses were conducted using SAS, version 9.2 (SAS Institute, Cary, NC, USA).

### Ethics statement

KNHANES VII was carried out following approval from the institutional review board of KCDC. All participants signed an informed consent form. The present study protocol conformed to the ethical guidelines of the 1975 Declaration of Helsinki, as revised in 1983. The Institutional Review Board of Samsung Medical Center approved the current study (number: SMC 2018-08-176).

## Supplementary information

Supplementary Table S1

## Data Availability

The datasets generated during the current study are available from the corresponding author upon reasonable request.
