# Supplementary material for: Association between anemia and hyperuricemia: results from the Korean National Health and Nutrition Examination Survey
Source: Sci Rep. 2019 Dec 13;9:19067. doi: 10.1038/s41598-019-55514-y (PMC6911023; doi:10.1038/s41598-019-55514-y)
Supplement: Supplementary file 1 — Supplementary Table S1 [file 41598_2019_55514_MOESM1_ESM.pdf]

Association between anemia and hyperuricemia: results from the Korean National Health and Nutrition Examination Survey

Yeonghee Eun[1], Kyung-Do Han[2], Da Hye Kim[2], In Young Kim[3], Eun-Jung Park[4], Seulkee Lee[1], Hoon-Suk Cha[1], Eun-Mi Koh[1], Jaejoon Lee[1,*,†], and Hyungjin Kim[1,*,†]

[1] Department of Medicine, Samsung Medical Center, Sungkyunkwan University School of Medicine, Seoul, Korea

[2] Department of Biostatistics, College of Medicine, The Catholic University of Korea, Seoul, Korea

[3] Department of Medicine, National Police Hospital, Seoul, Korea

[4] Department of Medicine, National Medical Center, Seoul, Korea

[*] Correspondence: jaejoonlee.lee@samsung.com (J.L.); passiondoc@gmail.com (H.K.)

[†] These authors contributed equally to the work.

Correspondence and request for materials should be addressed to Dr. Hyungjin Kim (E-mail: passiondoc@gmail.com)

Supplementary Table S1. Multivariate logistic regression analysis of the association between anemia and hyperuricemia when CKD is divided into two groups based on an eGFR of 30.

Subgroup	OR (95% CI)				
	Model 1	Model 2	Model 3	Model 4	Model 5
Total	0.68 (0.54-0.87)	1.18 (0.93-1.51)	1.42 (1.11-1.82)	1.44 (1.12-1.84)	1.18 (0.90-1.53)
Non-CKD	0.29 (0.21-0.42)	0.57 (0.39-0.83)	0.68 (0.47-0.99)	0.69 (0.48-1.01)	0.73 (0.50-1.10)
eGFR 30-60<br>(n = 308)	2.05 (1.44-3.67)	2.03 (1.13-3.66)	2.37 (1.22-4.61)	2.51 (1.25-5.04)	2.24 (1.10-4.56)
eGFR <30<br>(n = 33)	3.24 (0.43-24.52)	2.72 (0.35-21.17)	*	*	*

ORs and 95% CIs for the presence of hyperuricemia. Model 1 is non-adjusted model. Model 2 is adjusted for age and sex. Model 3 is adjusted for all variables in model 2 plus BMI, smoking, alcohol drinking, physical activity, income, education level. Model 4 is adjusted for all variables in model 3 plus hypertension and diabetes. Model 5 is adjusted for all variables in model 4 plus glomerular filtration rate. *Further multivariate logistic regression analysis was not possible due to the small size of this subgroup. OR, odds ratio; 95% CI, 95% confidence interval; CKD, chronic kidney disease, eGFR, estimated glomerular filtration rate.
